# AI usage and employee creativity: the mediating role of work engagement and the moderating role of technology acceptance

**DOI:** 10.3389/fpsyg.2026.1807038

**Published:** 2026-05-28

**Authors:** Han Cai, Zhiming Wang, Xiu Jin

**Affiliations:** 1 Department of Cross-border E-commerce, School of Management, Liaodong University, Dandong, China; 2 Department of Hotel and Tourism Management, Youngsan University, Yangsan-si, Republic of Korea; 3 Department of Business Administration, Gachon University, Seongnam, Republic of Korea

**Keywords:** AI usage, employee creativity, SMEs, technology acceptance, work engagement

## Abstract

In the new era characterized by the accelerated integration of science, technology, and industry, artificial intelligence (AI) is increasingly becoming a core force driving enterprise transformation. Innovation serves as the primary engine of rapid corporate development, with employee creativity being the key to such innovation. As an externally enabling technological tool, AI not only effectively extends the boundaries of employee creativity but also significantly unlocks employees’ innovative potential. Therefore, enhancement of employee creativity is closely related to the effective utilization of AI technology. Based on conservation of resources theory and social exchange theory, this study develops a research model to investigate how AI usage affects employee creativity. Moreover, previous studies have predominantly focused on modeling mediating effects. To address this research gap, the present study broadens its scope by incorporating the moderating role of technology acceptance and examining its effects. Targeting Chinese SMEs, the study collected 316 samples and performed empirical analyses using SPSS and Amos. The findings reveal that AI usage influences employee creativity through work engagement. In addition, the interaction between technology acceptance and AI usage amplifies the effects on both work engagement and employee creativity. Theoretically, this study advances interdisciplinary research by integrating AI technology with organizational behavior, thus establishing a foundation for subsequent studies. Practically, it provides concrete guidance for Chinese SMEs in their digital transformation endeavors.

## 1 Introduction

Against the backdrop of the new technological revolution and industrial change, artificial intelligence (AI) technology, as the core driving force, has received extensive attention from all walks of life and is being practiced and applied in depth (Wang et al., 2025). In recent years, this technology has flourished and has become a key force in the transformation of China’s traditional economy (Zhao and Tang, 2025). Moreover, owing to its remarkable characteristics, such as environmental adaptability and creativity, AI technology has gradually achieved a partial substitution of traditional production technology, effectively optimized enterprise processes, and provided powerful support for enterprise development in the new era (Chao and Shen, 2025). AI usage can help employees more actively embrace technological change within the organization, protect against potential losses, promote the reduction of risks, and provide technical support to manage current and future resources (Qian et al., 2025). Specifically, AI usage refers to the extent to which employees make their first choices at work (Joyner et al., 2024). At work, when employees are proficient in AI, they will be more proactive in identifying and selecting the most appropriate AI tools, learning efficiently with the help of AI technology, and thus supporting the further development of the organization (Jing et al., 2024). Moreover, the application of AI technology in the workplace can personalize workflows and achieve high efficiency, more accurately meeting the needs of the organization and improving its overall effectiveness and quality (He, 2025). Therefore, the key role of AI in organizational development has become increasingly prominent. In the context of the digital economy, enterprises should actively promote and strengthen the use of AI among employees, which not only helps them achieve efficient transformation in an environment full of uncertainty but also gives them a significant advantage and provides strong support for future competition in the market.

In the context of the rapid development of AI technology, organizations need to rely not only on technological power but also on employee intelligence to stand out in a complex and changing environment (Zhao and Tang, 2025). As the source of an organization’s core competencies, employee creativity—that is, the ability to come up with novel and practical ideas (Dharmanto and Rony, 2023)—is a core manifestation of employee wisdom and a key driver of organizational success. The extensive use of AI technologies in the workplace can provide employees with richer information resources and analytical tools, effectively stimulate new ideas, and significantly enhance employee creativity (Chen et al., 2025). This not only directly helps employees enhance their competence, knowledge, and practical experience (Nasir et al., 2022) but also efficiently contributes to the achievement of the organization’s strategic goals through innovative solutions. However, employee creativity does not exist in isolation; it is essentially the result of a complex interaction between individual cognitive factors (e.g., knowledge, skills, and mindset) and environmental factors (e.g., organizational climate, resource support, and leadership style; Peng et al., 2020; Zhou and Long, 2011). Therefore, when introducing AI tools to empower employees, organizations must understand that the organic combination of advanced technological tools and a humanistic environment that stimulates employees’ creative potential is the key to building a sustainable innovation advantage. By enhancing employee AI usage, we can not only increase the potential for employee innovation but also further enhance organizational creativity. Therefore, we predict that employee AI usage can enhance creativity and facilitate the future development of organizations.

This study aims to determine whether the use of AI contributes to the enhancement of employee creativity, thereby increasing the overall level of creativity among employees. This finding also suggests that work engagement mediates the relationship between AI use and employee creativity. AI usage can help employees better understand and respond to task characteristics while creating an environment that is more conducive to work engagement (Qian et al., 2025). Work engagement refers to the depth of focus and active participation that individuals demonstrate in the workplace (Li and Ling, 2007). A high level of work engagement is the perfect link between individual traits, work factors, and work performance, and it is an important way for organizations to create a competitive advantage (Hu and Wang, 2014). When employees are highly engaged in their work, they exhibit high energy and a strong sense of identity, which significantly improves work efficiency (Li and Ling, 2007). Moreover, when employees have a high level of work engagement and are less exposed to external distractions, they are better able to generate creative ideas and solve problems effectively, significantly enhancing their creativity (Wu et al., 2021). The appropriate use of AI can provide employees with high-quality resources to alleviate their fatigue when coping with workloads, keep them energized in the face of work demands, increase their work engagement, improve their focus on core job content, fully develop their potential, and promote personal creativity (Montani et al., 2020). Therefore, this study assumes that the use of AI can affect employee creativity through work engagement.

In addition, this study suggests that employee creativity and work engagement change because of the interaction between technology acceptance and AI usage. Therefore, this study examines technology acceptance as a moderating variable and verifies its interaction with AI usage to further understand their effects on employee creativity and work engagement. Technology acceptance refers to an individual’s willingness to actively choose and adopt new technologies (Türkyılmaz, 2023). When individuals perceive technology as being more useful and easier to use, they tend to have a more positive attitude toward it and a clearer intention to use it, which makes them more likely to adopt it (Xin and Guo, 2025). Moreover, when employees have a higher level of acceptance of AI-using technologies, they tend to have a more positive attitude toward AI use at work, which can enhance their positive emotions (Xin and Guo, 2025). Regarding employee creativity, when employees are more receptive to AI usage, they tend to be more willing and proactive in using AI at work. The cognitive resources and creative support generated by technology are positively associated with employees’ innovative thinking and creativity. Regarding work engagement, higher levels of technology acceptance within an organization tend to co-occur with employees’ greater willingness to integrate these technologies into their work, which may help reduce repetitive tasks. This shift in task allocation is related to employees focusing more on strategic and creative activities, and such focused attention is positively linked to their sense of meaning and engagement in work. Based on these considerations, exploring the moderating role of the Technology Acceptance Model (TAM) is theoretically meaningful. The interaction between technology acceptance and AI usage can enhance employee creativity and work engagement.

Based on the above theories, the purpose of the study is summarized as follows: First, AI, as a major achievement in the development of social science and technology, has become the core force driving a new round of industrial change (Xin and Guo, 2025). However, current research has paid relatively little attention to the interdisciplinary integration of AI usage and organizational behavior, particularly the lack of systematic empirical evidence on how AI usage affects employee creativity. Moreover, existing research typically views AI in the workplace as a factor that may deprive or diminish employee resources (Liu and Li, 2025). Therefore, this study explores the positive effects of AI in organizational contexts to fill this research gap. It focuses on the application of AI in the workplace, aiming to reveal the mechanisms underlying its influence on employees’ psychological states and work attitudes. This study aims not only to deepen the understanding of the internal psychological path of AI-empowered employees but also to provide an important theoretical basis and practical guidance for organizations to effectively utilize AI technology, optimize management practices, stimulate employees’ potential, and ultimately enhance organizational innovation.

Second, as an indispensable component of organizations, individual creativity is critical for organizational success in the digital era (Ouyang and Zhu, 2025; Wei and Wu, 2025). Given that creativity is a product of complex interactions between individual traits and situational factors, this study focuses on individual creativity—specifically, employee creativity—in the context of digital transformation.

Third, most existing studies emphasize how individuals accept and use new technologies through the TAM but neglect the impact of technology on individuals (Ozili, 2025). By contrast, this study highlights the impact of technology acceptance on individual organizations by identifying the moderating role of employee technology acceptance and validating its effect on the relationship between AI usage, work engagement, and employee creativity. Regarding moderation, we determined how the interaction effect influenced employee creativity and work engagement by examining the relationship between technology acceptance and AI usage.

Finally, although existing research has preliminarily explored the relationship between artificial intelligence (AI) usage and employee creativity, important research gaps remain regarding the underlying transmission mechanisms and boundary conditions of this relationship. The existing literature has largely focused on the direct effects of AI usage, while insufficient attention has been paid to work engagement as a key mediating process. Drawing on conservation of resources theory and social exchange theory, the efficient information processing and decision support provided by AI usage are more likely to help employees develop positive emotional investment in their work, thereby enhancing their level of work engagement and indirectly influencing employee creativity. However, this logical chain has not been directly tested in previous studies. Furthermore, in the digital work environment, employees’ level of technology acceptance not only affects the effectiveness of AI usage but also plays a critical role in enhancing employee work engagement and creativity through its interaction with AI usage—that is, it moderates the impact of AI usage on work engagement and employee creativity. Based on the above analysis, this study aims to reveal the mediating effect of work engagement in the relationship between AI usage and employee creativity, systematically examine how technology acceptance moderates the effects of AI usage on work engagement and creativity, and provide a more comprehensive explanation of how AI usage can effectively enhance employee creativity by integrating a theoretical framework that combines mediation and moderation. Ultimately, this study will reveal the extent of AI usage among employees in Chinese SMEs and provide insights to support future digital development.

## 2 Theoretical background and hypotheses

### 2.1 AI usage and work engagement

In the enterprise’s digital transformation stage, the improvement in employees’ work efficiency is closely related to AI usage. By integrating multiple types of applications and tools, AI can help employees solve problems at work more efficiently, allowing them to complete tasks more effectively (Jing et al., 2024). Specifically, AI usage refers to the level and extent to which employees use AI technology to accomplish their tasks (Man Tang et al., 2022). It also refers to the application of AI by employees to solve category-specific task problems at work (Liu and Wang, 2020). In addition, the use of AI can help employees simplify repetitive tasks in their work, thereby reducing resource consumption (Liu and Li, 2025). Moreover, based on prior research, AI usage has a positive impact on learners’ effectiveness in problem-solving using ChatGPT (Jing et al., 2024), employee performance (Prasad and De, 2024), and employee innovation behavior (Qian et al., 2025).

Work engagement is an important manifestation of employees’ concentration, positive emotions, and high cognitive engagement (Wang et al., 2012). This refers to the depth of concentration and positive engagement that an individual demonstrates at work (Geldenhuys et al., 2014). It also refers to the level of employee concentration at work (Gao and Zhan, 2021). A high level of work engagement leads to a greater focus on the employee’s psychological experience of the job and the work situation, and it promotes self-expression at work (Li and Ling, 2007). Employees with high work engagement are passionate about their work, proactively invest more time and energy, and focus more on it (Qin, 2024). In addition, according to a collation of prior research, transformational leadership (Gözükara and Simsek, 2016), perceived organizational support (Aldabbas et al., 2021), and work-family support (Wang et al., 2012) can positively impact employees’ work engagement.

According to resource conservation theory, when employees have sufficient resources, they can effectively reduce the consumption of work resources and more easily obtain additional resources. AI usage helps employees solve complex problems and increases their awareness of resource acquisition, which translates into psychological confidence and readiness, thereby enhancing work engagement (Liu and Li, 2025). In practice, AI usage can help employees combine intuition with data-driven decision-making, which further enhances their confidence and sense of control over their work, thereby promoting work engagement (Haider and Ali, 2024). In addition, when employees actively engage in critical thinking during the application of AI and thoroughly explore the methods and effectiveness of its use in work situations, they can fully stimulate their potential and further enhance their work engagement (Zhao et al., 2025). Simultaneously, to protect and accumulate resources more efficiently, employees actively utilize AI to enhance their capabilities, a process that also contributes to their work engagement (Zhao et al., 2025). Based on these considerations, this study proposes the following hypothesis:

*Hypothesis 1*: AI usage will positively influence work engagement.

### 2.2 AI usage and employee creativity

Employee creativity refers to the ability of employees to generate innovative and practical ideas at work (Tang et al., 2017), or more specifically, the ability to express novel ideas or propose new approaches (Bogar, 2023). As a core source of firm value, creativity drives firms to continuously innovate, enabling them to stay ahead of the curve and achieve sustainable growth in an increasingly competitive market (Nasifoglu Elidemir et al., 2020). Therefore, employee creativity is crucial for firms to continuously consolidate and enhance their level of innovation (Zheng et al., 2025), and is regarded as one of the most critical factors for organizations to gain and sustain competitive advantage (Kremer et al., 2019). Prior research found that perceived organizational support (Aldabbas et al., 2021), and challenge stress (Wu et al., 2021) have a positive impact on employee creativity.

Employee creativity is a core factor in organizational development. This study emphasizes the key role of AI. Specifically, AI has become an important technology to drive organizational progress in the digital economy (Liu et al., 2024). Employees have access to resource support for AI usage, which helps alleviate the anxiety associated with risk and uncertainty associated with innovative behaviors and promotes a reduction in perceived risk; they may be more inclined to pursue bolder innovations, thereby enhancing their own creativity (Qian et al., 2025). Moreover, the use of AI can help employees quickly cope with repetitive and difficult cognitive tasks at work, break the limitations of attention, and expand the scope of information search, thus stimulating employees’ intrinsic motivation to work, enhancing critical skills, and promoting creativity (Gao, 2025). AI usage helps enhance employees’ basic skills and accumulates rich knowledge, providing the necessary resources for creativity (Chen et al., 2025). Simultaneously, AI supports individuals in generating creative ideas by classifying, organizing, and analyzing large amounts of information and identifying patterns of association between them (Gao, 2025). Based on these considerations, this study proposes the following hypothesis:

*Hypothesis 2:* AI usage will positively influence employee creativity.

### 2.3 Work engagement and employee creativity

Employee creativity is a key factor in the survival and development of organizations. However, the effective stimulation of employee creativity faces significant challenges (Dong et al., 2017). In this context, high work engagement is an important means of stimulating creativity. Specifically, when employees demonstrate high levels of work engagement, they experience a strong sense of motivation, abundant energy, and a deep passion for their work (Aldabbas et al., 2021). This positive motivational state leads employees to be more proactive in pursuing valuable work goals and actively exploring complex tasks (Aldabbas et al., 2021). Highly engaged employees are more willing to undertake challenging tasks, face the risks associated with innovation, and proactively seek the best ways to achieve their goals (Qin, 2024). Thus, work engagement not only enhances employees’ ability and willingness to generate creative ideas (Aldabbas et al., 2021) but also makes it easier for employees to transform their own creativity potential into actual innovations (Wang et al., 2012), which ultimately improves their creativity performance at work. Based on these considerations, this study proposes the following hypothesis:

*Hypothesis 3*: Work engagement will positively influence employee creativity.

### 2.4 The mediating effect of work engagement

AI empowers efficient information processing and decision-making support, significantly improving employees’ motivation and ability to work, which leads to a more in-depth understanding of information and the formation of opinions. This deepening process not only enhances employees’ work engagement and enthusiasm but also strengthens their subjectivity in information presentation, resulting in a simultaneous increase in employees’ sense of responsibility, motivation, and ability to make decisions. Consequently, employees’ confidence in their work is enhanced, which significantly stimulates their creativity (Gao, 2025). Concurrently, the integration of AI into daily work not only helps employees to acquire new skills but also facilitates the flow of knowledge by breaking down information silos and building sharing platforms, thus enhancing employee work engagement, professionalism, and creativity (Ali et al., 2024). Notably, when organizations explicitly position AI at the core of their work and actively encourage its use, employees are more inclined to proactively adopt AI tools, recognize their significant benefits and job relevance, and view them as rich sources of information. Such positive acceptance effectively enhances work engagement and stimulates the potential for active exploration, thus becoming an important driver of creativity (Tong et al., 2025). Additionally, the application of AI not only provides employees with more resources to cope with workload growth but also enables them to immerse themselves deeper into the core of their work, manage complex tasks more effectively, and significantly reduce the burden of repetitive tasks through efficient information processing and analysis tools. The cognitive resources and time thus freed allow employees to explore new ideas and approaches, stimulate innovation, and ultimately enhance employee creativity (Montani et al., 2020). Equally critical, AI technology provides strong support for the continuous improvement of employee creativity by optimizing work design and division of labor, automating programmed tasks, and providing the necessary resources to motivate employees to engage in work, solve problems proactively, and explore actively (Xun, 2024). In addition, based on social exchange theory, when employees perceive organizational support through valuable resources like AI tools, they feel obligated to reciprocate with positive attitudes and behaviors. AI usage thus represents an organizational investment in employee productivity and well-being. Beneficiaries of such support tend to respond with greater work engagement, which in turn promotes creativity, as engaged employees are more motivated to contribute innovative ideas beyond basic job requirements. Based on these considerations, this study proposes the following hypothesis:

*Hypothesis 4*: Work engagement mediates the relationship between AI use and employee creativity.

### 2.5 The moderating effect of technology acceptance

This study emphasizes the moderating role of employees’ technology acceptance and suggests that its emergence will enhance the role of AI usage in employee creativity and work engagement. Thus, individual employee creativity and work engagement in Chinese SMEs are determined by the interaction between technology acceptance and AI usage. Technology acceptance refers to the willingness and extent to which employees use and adapt to new technologies (Musa et al., 2023). It also reflects employees’ willingness and motivation to use information technology to accomplish tasks (Teo and Van Schaik, 2012). When employees’ technology acceptance is high, they are more likely to feel a higher sense of accomplishment and success at work, which promotes positive work engagement and career development (Xin and Guo, 2025). Moreover, the adoption, implementation, and frequent use of new technologies are essential to promote an understanding of the need for change within an organization (Tayal et al., 2018). Prior research also demonstrated that employee acceptance of new technology has a positive impact on employees’ sense of job prosperity (Xin and Guo, 2025) and a negative impact on job burnout (Xin and Guo, 2025).

Employees tend to use AI because it provides more accurate and reliable information and advice, which enhances employees’ trust in it, increases their willingness to use it, and promotes its application in the organization (Jiang et al., 2024). Therefore, higher technology acceptance among employees enables them to quickly recognize the usefulness and ease of use of new technologies, effectively integrate information, and explore new approaches, which leads to more active adoption of new technologies and enhanced creativity at work (Putro and Takahashi, 2024). Moreover, when employees are more receptive to technology, they perceive the information they receive from new technologies as more valuable and are more likely to trust and emulate the use of these technologies, thus enhancing their creativity (Zhang et al., 2020). Concurrently, when employees are open and receptive to new technologies, they are more willing to learn and apply these technologies to explore new work methods and solutions, and this attitude of active exploration helps to stimulate innovative thinking, which enhances creativity (Itoe Mote and Karadas, 2022). In this process, AI not only helps employees integrate information efficiently and optimize the quality of decision-making through in-depth processing and analysis of information but also significantly improves their related skills, which ultimately enhances employee creativity (Gao, 2025). Therefore, when the level of technology acceptance was high, the impact of AI use on employee creativity increases. When employees demonstrate a higher level of technology acceptance, they are more willing to actively explore and apply AI tools, which not only improves work efficiency but also provides a wider range of knowledge and information resources. Through AI, employees can handle complex tasks more efficiently, and thus have more time and energy to devote to innovative activities. This virtuous cycle enhances employee self-confidence and work engagement, creates a supportive work environment for innovation, and significantly increases employee creativity.

Additionally, high levels of technology acceptance enable employees to access and apply AI technologies earlier and more easily in their work (He, 2025). When AI provides more accurate and reliable advice on the job than the employees themselves, it prompts employees to develop higher levels of trust in their advice, which reinforces their acceptance of AI (Jiang et al., 2024). This increased trust drives employees to use AI more extensively to solve work problems (Kang et al., 2024). Simultaneously, when new technologies are introduced into an organization, ensuring that employees are fully engaged and understand the reasons, processes, and impacts of the changes will lead to a sense of ease of acceptance, a feeling of protection of their resources, and ultimately, an increase in work engagement (Molino et al., 2020). In addition, technology acceptance, as a key resource, empowers employees to successfully communicate and collaborate across geographic boundaries, thereby effectively increasing their work engagement (Khoza et al., 2024). Simultaneously, AI usage can help employees deal with routine and repetitive tasks more efficiently at work, thus saving their mental resources, increasing their mental availability, and enhancing work engagement (Liu and Li, 2025). Therefore, when the level of technology acceptance is high, the impact of AI usage on employees’ work engagement increases. Specifically, a high level of technology acceptance enables employees to efficiently utilize AI to handle routine and repetitive tasks, thereby saving time and energy. These saved resources can then be reallocated to creative and challenging tasks, thereby increasing work engagement.

Overall, employees with high technology acceptance manage their mental resources more effectively when using AI, reduce fatigue from repetitive tasks, and devote more energy to work engagement. Therefore, this study used the technology acceptance as a moderating variable for AI usage, work engagement, and employee creativity. The moderating effect of AI usage on employee creativity and work engagement is emphasized. Based on these considerations, this study proposes the following hypotheses:

*Hypothesis 5*: Technology acceptance will have a positive moderating effect on the relationship between AI usage and employee creativity.

*Hypothesis 6*: Technology acceptance will have a positive moderating effect on the relationship between AI usage and work engagement.

## 3 Methods

### 3.1 Sample characteristics

This study focused on individuals working within Chinese SMEs and collected data using an online survey instrument (SoJump). In compliance with informed consent and data confidentiality principles, all participants reviewed the information pamphlet attached at the start of the questionnaire before participating in the study. This pamphlet informed participants that the research would be conducted anonymously and would not cause any disturbances or consequences to their organizations.

To collect data for our study, we used a snowball sampling technique to distribute our research questionnaire to members of SMEs within our professional network, focusing on individuals with whom we had established professional relationships. Specifically, we first identified SMEs across multiple industries as initial survey subjects and selected 2–3 core employees from each enterprise. We then asked these initial respondents to forward the questionnaire to their colleagues within the same enterprises, thereby expanding the scope of the survey.

Snowball sampling adopts the “acquaintance recommendation” model by forwarding questionnaires to peers within the industry, which reduces corporate resistance and provides more truthful and detailed feedback from enterprises. This approach yielded 316 responses from Chinese SMEs. All participants were assured of confidentiality to protect the integrity of the data and secure their cooperation. The data collected were strictly for the purposes of this study, with a firm commitment not to disclose them to any external party. However, snowball sampling has natural academic limitations: (1) the risk of non-representative samples. If the initial samples are concentrated in a specific region or industry, it may easily lead to homogenization of subsequent forwarded samples; (2) the hidden danger of sampling deviation, employees tend to send questionnaires to colleagues with similar perceptions (departments and positions).

A power analysis was conducted using G*Power 3.1 software (Faul et al., 2007). The analysis established a significance level of *α* = 0.05, indicating statistical significance, and identified an effect size of *f* = 0.15, which reflects a moderate effect. Employee creativity was the primary predictor. The total number of predictors included three components: the independent variable (X), mediating variable, and moderating variable, which corresponded to AI usage, work engagement, and technology acceptance, respectively. Power analysis revealed that a minimum sample size of 89 was required to achieve a statistical power of 95%. To ensure a sufficient dataset for analysis, this study ultimately included a sample size of 316 participants, which significantly surpassed the 89 participants recommended by the power analysis. Therefore, the sample size for this research was considered highly adequate and met the standards for thorough analysis.

Regarding demographic information, 152 participants (48.1%) were men and 164 (51.9%) were women. One participant (0.3%) was under 20-years-old, 127 (40.2%) were aged 20- to 30-years-old, 77 (24.4%) were aged 31- to 40-years-old, 59 (18.7%) were aged 41- to 50-years-old, 49(15.5%) were aged 51- to 60-years-old, and three (0.9%) were aged 61 years or older.

Regarding education, 105 (33.2%) were junior college graduates or lower, 126 (39.9%) were college graduates, 62 (19.6%) had master’s degrees, and 23 (7.3%) held a doctoral degree.

Regarding service years, 53 (16.8%) had worked for less than 1 year, 59 (18.7%) for 1 to 2 years, 28 (8.9%) for 3 to 4 years, 29 (9.2%) for 5 to 6 years, and 147 (46.5%) for seven or more years.

Regarding enterprise type, 46 (14.6%) worked in the construction category, 90 (28.5%) in catering services, 36 (11.4%) in finance, 26 (8.2%) in education, 17 (5.4%) in the medical industry, 26 (8.2%) in the information technology industry, and 75 (23.7%) in other occupations.

### 3.2 Measurement

Artificial intelligence(AI) usage refers to the process by which employees complete professional tasks and improve work efficiency in practice by leveraging AI tools or technologies (Yildirim and Şahin, 2026). To measure Chinese SMEs’ AI usage, this study used a tool mentioned by Yam et al. (2023), which comprises four items. Sample items included “Today, many of the decision-making activities of this job were automated or assisted by robots” and “Today, many problem-solving activities in this job are automated or assisted by robots.” It should be noted that the original scale used in this study included the term “robots.” When administering the questionnaire, we explicitly defined “robots” within the Chinese cultural context as AI tools capable of assisting with work tasks, such as information processing, decision support, and problem-solving. Therefore, when respondents answered the relevant items, their understanding of “robots” was not limited to traditional physical robots but extended to a broader range of AI applications.

Work engagement refers to employees’ sense of identity and emotional connection with their work. It emphasizes their work and the degree of concentration they are willing to devote to it (Gözükara and Simsek, 2016). To measure Chinese SMEs’ work engagement, this study used the tool mentioned by Schaufeli et al. (2006). The measurement tool comprises nine items. Sample items included “At my job, I feel strong and vigorous” and “I am enthusiastic about my job.”

Technology acceptance refers to employees’ psychological tendencies and attitudes toward voluntarily or proactively using a particular technology (Chen B. et al., 2024; Chen K. J. et al., 2024). This study used the tool mentioned by Davis (1989) to measure Chinese SMEs’ technology acceptance. The measurement tool consisted of 12 items. Sample items included “AI technologies would help me complete tasks faster” and “Learning AI technologies would be easy for me.”

Employee creativity refers to employees’ ability to generate novel and practical ideas or solutions at work (Nasifoglu Elidemir et al., 2020). To measure employee creativity among Chinese SMEs, this study adopted the four-item scale of Jaiswal and Dhar (2015), which features concise and clear items that facilitate implementation in corporate settings and better align with the measurement needs of employee samples in the Chinese cultural context. Sample items include “This subordinate identifies opportunities for new ways of dealing with work” and “This subordinate seeks new ideas and ways to solve problems.” In addition, the original items of the scale used in this study were phrased in the third person singular (e.g., “This subordinate…”). To adapt the scale for employee self-report, we revised the items by replacing “This subordinate” with “I” in each item. For example, the original item “This subordinate identifies opportunities for new ways of dealing with work” was adjusted to “I identify opportunities for new ways of dealing with work.” This adaptation is widely used in self-report studies in organizational behavior.

All items were assessed using a seven-point Likert scale, with one representing “strongly disagree” and seven indicating “strongly agree.”

### 3.3 Statistical analysis

In this study, the analytical process was meticulously designed and carried out in the following sequence: Initially, power analysis was conducted using G*Power 3.1. This was followed by demographic analysis. Subsequently, correlation analysis and reliability analysis was performed using SPSS 23.0, and CFA was conducted using AMOS 24.0.

Mediation hypotheses were tested using the SPSS PROCESS Macro v3.4.1 Model 4, while moderation hypotheses were examined with the SPSS regression analysis. Table 1 is a definition table for all symbols in the study, which clarifies the meaning of some symbols and serve as a reference for symbols in this study.

**Table 1:** Symbol definitions in the study.

Symbol category	Specific symbol/expression	English meaning
Model and statistical symbols	*R* ^2^	Coefficient of determination
	Δ*R*^2^	Incremental explained variance
	F	F-statistic
	df (df1/df2)	Degrees of freedom (Numerator DF/Denominator DF)
	*p*	Significance Level
	*B*	Unstandardized regression coefficient
	*SE*	Standard error
	*t*	*t*-statistic
	LLCI/ULCI	Lower Limit of Confidence Interval/Upper Limit of Confidence Interval
Grouping and plotting symbols	+1 Std Dev	Mean +1 Standard Deviation
	−1 Std Dev	Mean −1 Standard Deviation
Significance markers	***	Mark for Extremely Significant Effect
	**	Mark for Significant Effect
	*	Mark for Marginally Significant Effect

**Figure 1:**
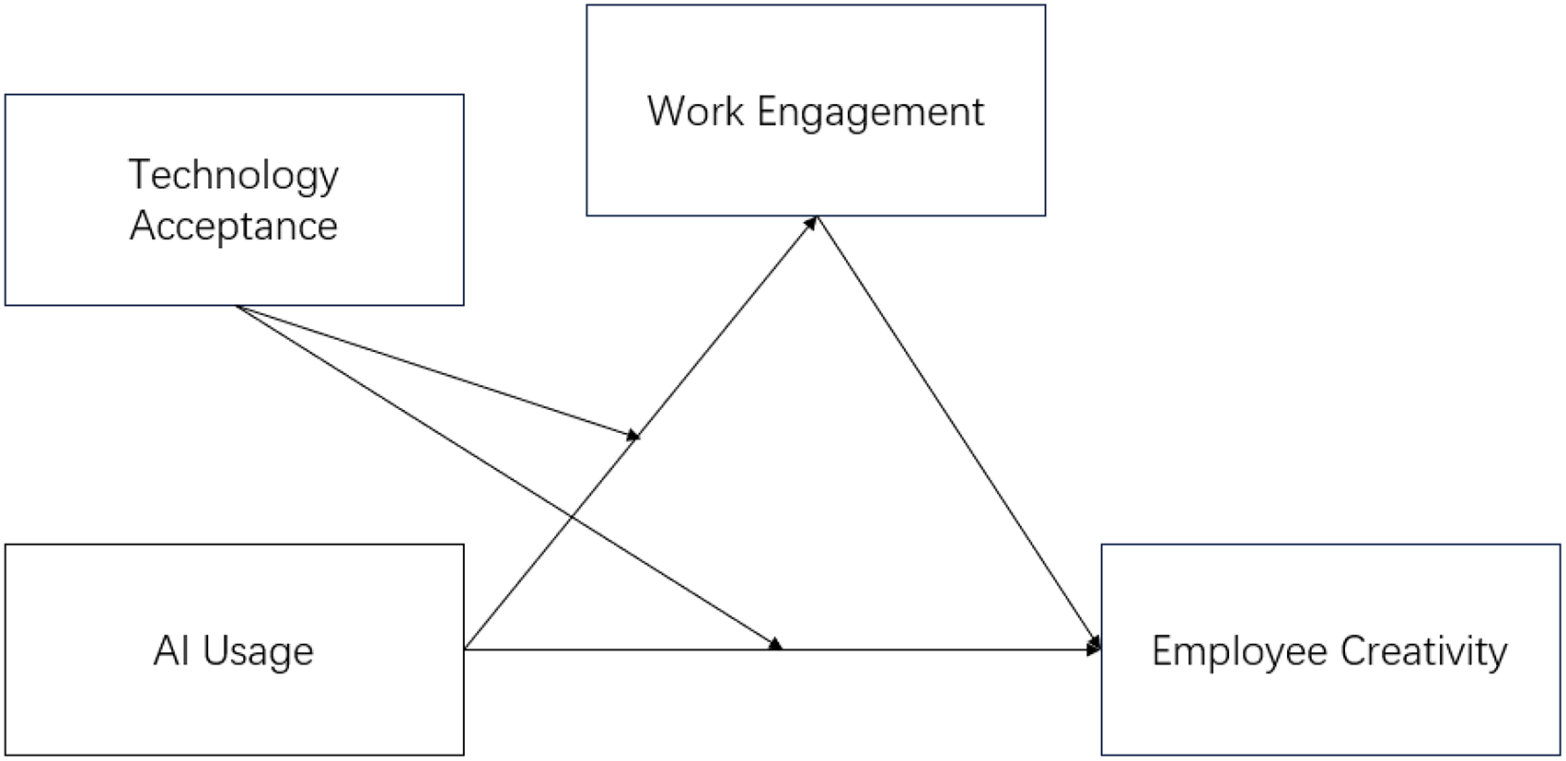
Research model.

In summary, SPSS23.0 is suitable for demographic analysis, reliability test, and descriptive statistics and correlation analysis of variables in this study, and efficiently completes basic statistics and preliminary validity test tasks. AMOS 24.0 has significant advantages in covariance structural equation modeling. It accurately carries out CFA to meet the needs of scale structure verification (see Figure 1).

## 4 Results

### 4.1 Confirmatory factor analysis and reliability analysis

In this study, the practicability of different data models is verified by confirmatory factor analysis (Presson et al., 1997). The results of confirmatory factor analysis are as follows. The absolute fit indexes were *χ*^2^ (*p*) = 1020.369(0.000), *χ*^2^/df = 2.721, and RMSEA = 0.074. And the RMSEA is indeed a “badness of fit” index, with values very close to 0 indicating almost perfect fit and with greater RMSEA indicating worse fit. For the RMSEA, values less than 0.05 reflect a small approximation error, values between 0.05 and 0.08 reflect an acceptable error of approximation while values greater than 0.10 constitute poor fit of the model (Browne and Cudeck, 1992). Second, the incremental fit indexes were IFI = 0.937 and CFI = 0.937. Third, the parsimonious adjusted indexes were PNFI = 0.835 and PGFI = 0.677.

This study analyzed the values of average variance extracted (AVE) and composite reliability (C.R). Regarding average variance extracted (AVE), AI usage was 0.674, work engagement was 0.658, technology acceptance was 0.674 and employee creativity was 0.689; these values were all greater than 0.5.

Regarding composite reliability (C.R), AI usage was 0.804, work engagement was 0.905, technology acceptance was 0.905 and employee creativity was 0.848; all these values were greater than 0.7. The measurement has significant validity if the AVE of variables is higher than 0.5 and CR is higher than 0.7.

Reliability analysis refers to the method of measuring the internal consistency of scale items (Qureshi et al., 2023). Therefore, this study also analyzed the Cronbach’s value. For values of *α* Cronbach’s, AI usage = 0.933, work engagement = 0.959, technology acceptance = 0.972 and employee creativity = 0.926; reliability analysis has significant validity if Cronbach’s of variables is higher than 0.7. The results are shown in Table 2.

**Table 2:** The result of confirmatory factor analysis and reliability analysis.

Variables	Estimate		S.E.	C.R.	*p*	Standardized regression weights	AVE	C.R	Cronbach’s alpha
AI usage	A4	1			***	0.749	0.674	0.804	0.933
	A3	1.201	0.05	23.817	***	0.863			
	A2	1.267	0.053	24.118	***	0.869			
	A1	1.187	0.056	21.072	***	0.797			
Work engagement (B)	B1	1				0.812	0.658	0.905	0.959
	B2	1.068	0.039	27.641	***	0.864			
	B3	1.027	0.041	25.319	***	0.833			
	B4	1.113	0.042	26.745	***	0.853			
	B5	1.073	0.053	20.305	***	0.745			
	B6	1.162	0.054	21.481	***	0.769			
	B7	1.092	0.043	25.324	***	0.834			
	B8	1.139	0.047	24.033	***	0.814			
	B9	1.007	0.047	21.581	***	0.771			
Technology acceptance (C)	C12	1				0.809	0.674	0.937	0.972
	C11	1.138	0.044	25.878	***	0.834			
	C10	1.105	0.042	26.333	***	0.84			
	C9	1.016	0.045	22.542	***	0.781			
	C8	1.053	0.044	23.811	***	0.803			
	C7	1.089	0.042	25.65	***	0.831			
	C6	1.091	0.042	25.919	***	0.836			
	C5	1.04	0.042	24.573	***	0.817			
	C4	0.967	0.04	24.144	***	0.81			
	C3	1.063	0.04	26.35	***	0.843			
	C2	1.01	0.043	23.579	***	0.801			
	C1	1.092	0.041	26.516	***	0.846			
Employee creativity (D)	D1	1			***	0.825	0.689	0.848	0.926
	D2	1.037	0.041	25.207	***	0.823			
	D3	1.125	0.041	27.236	***	0.85			
	D4	1.026	0.041	25.21	***	0.824			
Model fit index		X^2^(p) = 1020.369(0.000), X^2^/df = 2.721, RMSEA = 0.074, IFI = 0.937, CFI = 0.937, PGFI = 0.677, PNFI = 0.835							

### 4.2 Descriptive statistics and correlation analysis

Table 3 shows the descriptive statistics and correlation analysis. Descriptive statistics analysis included the mean and standard deviation (SD). The means for AI usage, work engagement, technology acceptance and employee creativity were 4.896, 5.057, 5.201 and 5.250, respectively. In addition, the SDs of AI usage, work engagement, technology acceptance and employee creativity 1.519, 1.298, 1.269 and 1.289, respectively.

**Table 3:** The results of descriptive statistics and correlation analysis.

Variables	Mean	Standard deviation	AI Usage	Work engagement	Technology acceptance	Employee creativity
AI Usage	4.896	1.519	—			
Work engagement	5.057	1.298	0.361^***^	—		
Technology acceptance	5.201	1.269	0.413^***^	0.804^***^	—	
Employee creativity	5.250	1.289	0.333^***^	0.852^***^	0.881^***^	—

***p < 0.001, **p < 0.01, *p < 0.05.

To verify the correlation among variables, this study conducted a correlation analysis; the results are summarized as follows: AI usage was positively associated with work engagement (*r* = 0.361, *p* < 0.001), technology acceptance (*r* = 0.413, *p* < 0.001) and employee creativity (*r* = 0.333, *p* < 0.001). Work engagement was positively associated with technology acceptance (*r* = 0.804, *p* < 0.001), and employee creativity (*r* = 0.852, *p* < 0.001). Moreover, technology acceptance was positively associated with employee creativity (*r* = 0.881, *p* < 0.001).

### 4.3 Hypothesis test

For hypothesis testing, this study employed Model 4 using the SPSS PROCESS Macro version 3.4 for analysis. The results show that AI usage has a positive impact on work engagement (Estimate = 0.3081, *p* < 0.001). But AI usage has not positive impact on employee creativity (Estimate = 0.0252, *p* > 0.05). In addition, the results show that work engagement has a positive impact on employee creativity (Estimate = 0.8358, *p* < 0.001). As a result, Hypothesis 1 and Hypothesis 3 were supported. But, hypothesis 2 was rejected.

Hypothesis 4 established that work engagement mediated the relationship between AI usage and employee creativity. The indirect effect was 0.257. The bootstrapped confidence intervals were Boot LLCI = 0.172 and Boot ULCI = 0.349, as 0 was not included between Boot LLCI and Boot ULCI. These results indicate that the mediation effect of work engagement was significant. This finding suggests that AI usage increased employee creativity through work engagement. Thus, Hypothesis 4 is supported. The Table 4 showed the results of path analysis.

**Table 4:** The results of process model 4.

Path			Estimate	S.E.	*t*	*p*	LLCI	ULCI
AI Usage	→	Work Engagement	0.3081	0.0450	6.8504	0.000	0.2196	0.3966
AI Usage	→	Employee Creativity	0.0252	0.0268	0.9380	0.349	−0.0276	0.0780
Work Engagement	→	Employee Creativity	0.8358	0.0314	26.6090	0.000	0.7740	0.8976
Indirect effect(s) of X on Y								
Indirect Effect			Effect	Boot SE	Boot LLCI		Boot ULCI	
AI Usage→ Work Engagement→ Employee Creativity			0.2575	0.0449	0.1729		0.3493	

### 4.4 Moderating effect of technology acceptance

**Figure 2:**
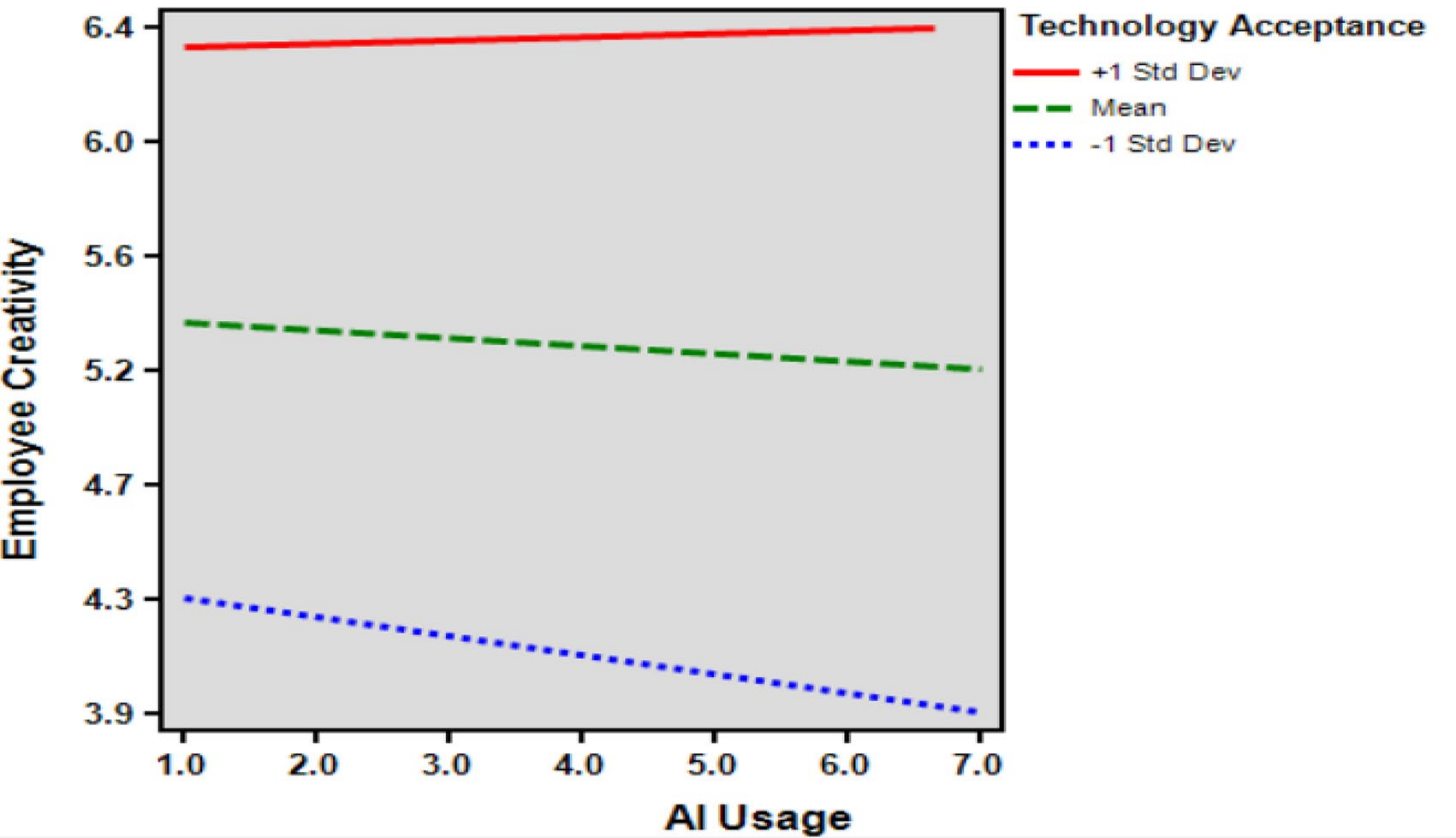
The moderating effect of technology acceptance.

Hypothesis 5 established that technology acceptance moderated the effect of AI usage on employee creativity. The results showed that technology acceptance significantly moderated the effect of AI usage on employee creativity (*β* = 0.064, *p* < 0.05). Hypothesis 5 proposed that technology acceptance moderates the effect of AI usage on employee creativity. The results showed that technology acceptance significantly moderates the effect of AI usage on employee creativity (*β* = 0.064, *p* < 0.05). However, in Figure 2, the slope corresponding to the mean level of technology acceptance appears to be negative, and the slope at −1 SD of technology acceptance appears even more negative. Therefore, to clarify the direction of the moderating effect, we conducted simple slope tests. The results (see Table 5) revealed that when technology acceptance was low (−1 SD), AI usage was significantly and negatively associated with employee creativity (*β* = −0.070, *SE* = 0.031, *p* = 0.027, 95% CI [−0.132, −0.008]); at mean levels of technology acceptance, the association was not significant (*β* = −0.029, *SE* = 0.025, *p* = 0.247, 95% CI [−0.077, 0.020]); when technology acceptance was high (+1 SD), the association was also not significant (*β* = 0.013, *SE* = 0.033, *p* = 0.703, 95% CI [−0.052, 0.077]). These findings suggest that technology acceptance does not simply amplify the positive effect of AI usage. Rather, the primary role of high technology acceptance is to attenuate or eliminate the negative association between AI usage and creativity observed under low technology acceptance conditions. In other words, AI usage is not negatively associated with creativity only when employees have sufficiently high levels of technology acceptance; under low acceptance conditions, AI usage may even be negatively related to creativity. Notably, although the specific pattern of the moderating effect differs from the initial hypothesis, the interaction effect is statistically significant, and the simple slope analysis reveals the differential impact of technology acceptance at different levels on the relationship between AI usage and employee creativity. Therefore, from a statistical perspective, Hypothesis 5 is supported (see Table 6).

**Table 5:** The result of moderation.

Dependent variable: employee creativity							
Variables	Model 1		Model 2		Model 3		
	*β*	*t*	*β*	*t*	*β*	*t*	VIF
AI usage (A)	0.333^***^	6.262	−0.036	−1.241	−0.034	−1.160	1.207
Technology acceptance (B)			0.896^***^	30.569	0.910^***^	30.351	1.273
Interaction					0.056^*^	2.019	1.081
*R^2^*(Adjusted *R^2^*)	0.111(0.108)		0.777(0.776)		0.780(0.778)		
*⊿R^2^*(⊿Adjusted *R^2^*)	-		0.666(0.668)		0.003(0.002)		
*F*	39.216^***^		545.135^***^		368.355^***^		

***p < 0.001, **p < 0.01, *p < 0.05.

**Table 6:** Simple slope.

Moderator	Level	Conditional effect	Boot SE	*t*	*p*	Boot LLCI	Boot ULCI
Technology acceptance	−1 SD (−1.269)	−0.070	0.031	−2.227	0.027	−0.132	−0.008
	M	−0.029	0.025	−1.160	0.247	−0.007	0.020
	+1 SD (1.269)	0.013	0.033	0.382	0.703	−0.052	0.077
Interaction: AI × technology acceptance							
	Estimate	Boot SE		*t*	*p*	Boot LLCI	Boot ULCI
	0.032	0.016		2.019	0.044	0.001	0.064

**Figure 3:**
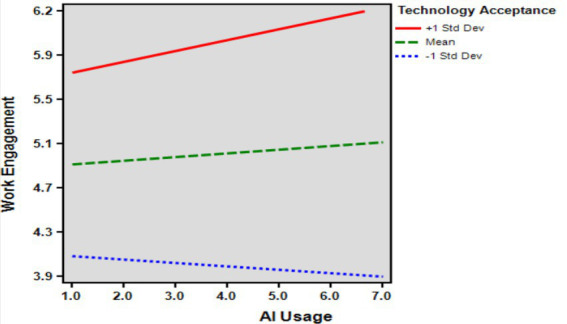
The moderating effect of technology acceptance.

Hypothesis 6 established that technology acceptance moderated the effect of AI usage on work engagement. The results showed that technology acceptance significantly moderated the effect of AI usage on work engagement (*β* = 0.084, *p* < 0.05). This means that the higher the technology acceptance, the greater the impact of AI usage on work engagement. Therefore, hypothesis 6 is supported. Therefore, through the results, we can find that the interaction between technology acceptance and AI usage will lead to a higher degree of work engagement (see Figure 3; Table 7).

**Table 7:** The result of moderation.

Dependent variable: work engagement							
Variables	Model 1		Model 2		Model 3		VIF
	*β*	*t*	*β*	*t*	*β*	*t*	
AI usage (A)	0.361^***^	6.850	0.035	0.948	0.039	1.057	1.207
Technology acceptance (B)			0.789^***^	21.416	0.811^***^	21.562	1.273
Interaction					0.084^*^	2.439	1.081
*R^2^*(Adjusted *R^2^*)	0.130(0.127)		0.647(0.645)		0.654(0.650)		
*⊿R^2^* (⊿Adjusted *R^2^*)	—		0.517(0.518)		0.007(0.005)		
*F*	46.928^***^		286.988^***^		196.332^***^		

***p < 0.001, **p < 0.01, *p < 0.05.

## 5 Discussion

According to previous studies (e.g., Acar, 2023; Wang et al., 2023; Xu et al., 2024), the important role of AI usage in organizations has been emphasized and confirmed. It is believed that increasing employee usage of AI in organizations is not only a key factor in improving employee creativity but also an important task in achieving sustainable organizational development and enhancing competitive advantage in organizational development. This study argues that the use of AI has emerged as the core of a new round of corporate reform due to social and technological developments. In addition, since employees are the main driving force behind enterprise development, this study focuses on the employees of Chinese SMEs and explores the impact of AI usage on them and the subsequent benefits it brings to the organization. Employee creativity reflects an individual employee’s ability to generate novel and innovative ideas. Therefore, this study considers employee creativity as a product of employees’ AI usage. The relationship between AI usage and employee creativity is also clarified. The current results suggest that AI usage can help employees provide quality resources in the organization, alleviate the fatigue of coping with workloads, reduce time costs, prompt employees to engage more deeply in their work, improve their understanding and observation of their tasks, and enhance their level of personal creativity.

### 5.1 Theoretical implications

The main contribution of this study is that it determined how the use of AI contributes to employee creativity. Not only does it focus on the direct impact of AI on employee creativity, but it also specifically explores which key variables play critical roles in this process. The induction of employee creativity plays a key role in the use of AI.

First, the AI usage has a positive impact on work engagement. This indicates that the higher the level of AI usage, the greater the level of employee work engagement. AI usage at work can help employees reduce work fatigue, increase job satisfaction and motivation, and enhance psychological availability (Liu and Li, 2025). Simultaneously, the application of AI technology can significantly improve the productivity and product quality of enterprises and enhance their market competitiveness (He, 2025). Consequently, AI usage can enhance employees’ self-confidence in work engagement, making them more certain of their abilities and status, which improves their psychological availability and promotes work engagement (Liu and Li, 2025). Higher levels of AI usage are related to employees’ improved ability to manage complex tasks, adjust work rhythm, and clarify priorities and goals, and this is associated with greater work engagement. While existing research has confirmed the positive influence of traditional job resources—such as perceived organizational support—the role of AI as a technological resource has remained largely under explored. The present study addresses this research gap by showing that, within Chinese SMEs, higher levels of AI usage and greater work engagement are positively related. This finding identifies AI usage as a distinctive contextual resource in the digital age, thus contributing a new theoretical perspective to the work engagement literature.

Second, while Zong et al. (2025) reported a positive effect of AI usage on creativity, the current study finds no direct effect of AI usage on employee creativity. This non-significant finding indicates that the level of AI usage does not directly determine employee creativity, as employee creativity emerges from the interaction between individual attributes and the work environment. AI, as a powerful external tool, excels at processing, analyzing, and supporting decision-making (Zhao et al., 2025), but serves only as an auxiliary tool that optimizes working conditions and provides resources. Employee creativity, by contrast, is an intrinsic ability requiring individual subjective cognition and environmental support. When both conditions are met, employees experience richer positive emotions, accumulate psychological resources, and gain impetus for creative thinking (Qin, 2024). Thus, AI usage does not directly enhance creativity. This non-significant direct effect challenges the assumption in existing literature that technological tools directly influence creativity. Our findings suggest that AI usage functions more as digital conditional empowerment, indirectly affecting employees by optimizing work conditions rather than directly boosting creativity. By demonstrating that AI usage influences creativity only indirectly through psychological mechanisms like work engagement, this study deepens theoretical understanding of the AI-creativity relationship. By clarifying the boundary between technological empowerment and individual cognitive processes, we show that external tools such as AI require internal psychological states to generate creative outcomes, contributing to the literature and offering a more nuanced perspective for future research on AI-driven innovation.

Third, work engagement positively affects employee creativity. This finding suggests that the higher the level of employees’ work engagement, the greater their personal creativity. Work engagement is a positive and deep state of integration, as demonstrated by individuals at work (Hu and Wang, 2014). It not only reflects the individual psychological state of employees but also serves as a core element of team and organizational success (Liu and Li, 2025). Employees with high levels of work engagement accumulate an abundance of positive emotions, as well as psychological and social resources. These resources provide support for employees to cope with challenging demands and creative risks, and they are an important basis for stimulating their creativity (Qin, 2024). Employees with a strong sense of engagement tend to invest more time and energy in work tasks, actively seek new paths and processes, and their tendency to generate novel ideas is positively associated with creativity.

Fourth, work engagement has a complete mediating effect on the relationship between AI usage and employee creativity. This finding suggests that the AI usage influences employee creativity through work engagement. As an evolving and innovative technology, AI has led to the change and development of many modern organizational practices (Haider and Ali, 2024). When employees can properly use and integrate AI, it not only helps them cope with technological challenges but also enhances their own competencies and skills, contributing to the development of the organization (Liu and Li, 2025). Moreover, as the use of AI in the workplace continues to increase, its impact on work engagement becomes increasingly significant, playing a crucial role in optimizing human resource management and enhancing the productivity and competitiveness of the organization (Liu and Li, 2025). AI usage can help employees recognize their potential strengths and strong connections to their work, which can increase their engagement, promote their understanding and observation of their tasks, and enhance their personal creativity (Tong et al., 2025). Therefore, based on the findings, this study suggests that work engagement is not merely a positive reaction to the work environment but also an intrinsic motivational mechanism that actively shapes creative outcomes. From the perspective of conservation of resources theory, employees with higher levels of work engagement generate greater resource returns, which in turn further stimulates creative thinking. Unlike previous studies that have largely focused on the direct effects of work engagement, the present study conceptualizes work engagement as a key mediating role between technological resources and individual creativity. This theoretical extension helps explain why employee creativity performance may differ across organizations that possess similar advanced technological tools, thereby providing theoretical support for the digital empowerment of enterprises.

Fifth, this study verifies the moderating role of technology acceptance in the relationship between AI use and employee creativity. The results showed that technology acceptance positively moderates the relationship between AI usage and employee creativity. However, simple slope analysis revealed that when the level of technology acceptance is low, AI usage is significantly negatively correlated with creativity; when technology acceptance is high, this negative association disappears. This finding suggests that technology acceptance does not simply “amplify” the positive effects of AI usage on employee creativity but rather “buffers” its potential adverse effects. In other words, under conditions of low technology acceptance, employees may experience negative emotions due to distrust or discomfort with AI tools, thereby inhibiting creativity. In contrast, high technology acceptance eliminates this negative effect by enhancing employees’ trust and positive attitudes toward AI. Technology acceptance influences employees’ perceptions and expectations of AI, which leads to positive use, encourages reuse, and creates conditions that prioritize AI usage (Kang et al., 2024). Therefore, employees are more inclined to actively experiment with and adopt these new technologies. This leads to increased beliefs and expectations, which can motivate them to further explore and utilize new technologies, supporting improvements in productivity and innovation (Khoza et al., 2024). This finding extends the technology acceptance literature by providing a new theoretical perspective for understanding under what conditions AI usage does not harm creativity. In summary, the findings regarding this moderating effect offer important theoretical contributions. This study identifies technology acceptance as a key boundary condition that determines the strength of the relationship between AI usage and creativity, thereby extending prior research that has primarily focused on the direct effects of technology acceptance. Furthermore, by demonstrating that technology acceptance can amplify the creative benefits of AI usage, this study bridges the technology acceptance literature with creativity research, providing an integrated theoretical perspective on human-technology interaction in the workplace. These findings reveal that fostering employees’ technology acceptance is not merely about facilitating technology adoption but also serves as an important mechanism for unlocking the creative potential of AI investments.

Finally, this study verifies the moderating role of technology acceptance in the relationship between AI usage and work engagement. The results showed that technology acceptance has a positive moderating effect on AI usage and work engagement. This indicates that higher levels of interaction between technology acceptance and AI usage are associated with greater levels of work engagement. Technology acceptance reflects employees perceived evaluation of the value of a new technological tool and their subjective assessment of the enhancement of work performance, leading to trust in AI usage (Lin et al., 2025). Employees are more likely to accept and use technology when they find it easy to use and helpful in performing their job-related duties. This acceptance increases their motivation and engagement (Khoza et al., 2024). Employees with a higher acceptance of technology are more proactive in integrating AI into their work. This allows them to efficiently handle repetitive tasks, reduce burnout, focus on their core responsibilities, and increase their task engagement. Therefore, this finding extends the technology acceptance literature by elucidating the significant role of technology acceptance in reinforcing the positive relationship between AI usage and work engagement. Through the identification of this moderating mechanism, the present study offers a more refined theoretical account of how AI usage facilitates enhancements in employee work engagement. This insight advances extant research by specifying the key moderating function of technology acceptance in the linkage between AI usage and work engagement, thus providing a novel perspective for understanding the micro-level mechanisms underlying human-AI collaboration.

In summary, the primary theoretical contribution of this study lies in conceptualizing AI usage as a novel technological job resource within the framework of conservation of resources theory. Unlike traditional resources such as organizational support or leadership, AI as a technological resource fundamentally reshapes the way employees interact with their work environment by optimizing task processes, reducing cognitive load, and enhancing decision-making efficiency. This study expands the boundaries of job resource research and provides a new theoretical perspective for understanding employee empowerment in technology-driven workplaces. Furthermore, this study identifies work engagement as a key mediating mechanism through which AI usage translates into employee creativity, and demonstrates that technology acceptance serves as an important boundary condition that moderates the effectiveness of AI usage. Taken together, these findings construct a comprehensive theoretical framework that not only explains “how” AI usage enhances employee outcomes (through work engagement) but also clarifies “when” it is most effective—specifically, under conditions of high technology acceptance—thereby providing theoretical support for future research.

### 5.2 Practical implications

First, with the deep integration of generative information technology and traditional industries, AI has gradually become a key factor in enhancing the overall effectiveness of enterprise innovation chains (Chao and Shen, 2025). With its popularization, employees can share knowledge directly with machines and transfer it through AI platforms (Malik et al., 2022). Knowledge sharing is effectively facilitated by streamlining workflows, which helps employees redirect their time and energy toward strategic work, creates a culture of continuous learning and collaboration within the organization, and plays a key role in facilitating the flow of expertise (Olan et al., 2024). AI usage can help employees access more resources while reducing time and costs, as well as increasing work efficiency. Therefore, in management practice, organizations should pay attention to employees’ level of AI usage and actively provide them with the necessary training and support to help them better accept and apply new technologies. In this way, the organization can not only improve the work efficiency of employees and access to resources but also aid its future development.

Second, employee engagement is an important factor in organizational management and productivity (Prasad and De, 2024), and employee work engagement is more a reflection of employee engagement at the operational level. According to the current results, work engagement has a fully mediating effect on AI usage and employee creativity. Specifically, AI usage has no direct effect on employee creativity; it only influences creativity through work engagement. This further confirms the importance of work engagement in organizations. Moreover, a high level of work engagement promotes employee creativity (Qin, 2024). Therefore, in future management practices, organizations should pay more attention to improving employee work engagement and ensure a positive and supportive working environment by optimizing the organization. By providing employees with the necessary resources and assistance in their work, employees can reduce the time cost of repetitive tasks and devote more energy to their core work engagements.

Third, as an important part of an organization, employees’ individual creativity is directly related to the organization’s innovation capabilities (Chen K. J. et al., 2024). Higher levels of employee creativity can enhance intrinsic motivation and the desire for organizational change, allowing employees to combine their ideas with their work to generate innovative behaviors (Du and Jin, 2023). Therefore, in future management practices, organizations should actively encourage employees to express themselves freely in their work. By fostering a positive organizational atmosphere, they can strengthen employees’ sense of organizational identity and provide effective psychological support, thereby stimulating employee creativity and offering robust support for the organization’s sustainable development.

Fourth, technology acceptance reflects an individual’s willingness and behavioral intention to actively engage in and adopt new technologies (Hadjiliasi et al., 2024). When employees have a high level of AI acceptance, they view AI collaboration as an opportunity for personal development rather than as a threat. This positive outlook enables them to face the challenging pressures of AI constructively, resulting in a greater sense of fulfillment and happiness at work (Xin and Guo, 2025). High technology acceptance can amplify the value of technology, reduce employee costs, improve work efficiency, and promote organizational advancements. Therefore, in future management practices, organizations should actively introduce AI or digital systems, encourage and advocate the acceptance of new technologies and work, and help employees improve their work efficiency. In addition, organizations should conduct regular training to emphasize the development of technology for personal development, help employees who are resistant to accepting it, and achieve a successful transformation for the organization in the digital stage.

### 5.3 Limitations and future research

Although this study validates the impact of work engagement on the relationship between AI usage and employee creativity, as well as the moderating role of technology acceptance, it has some limitations.

First, it focuses on the positive impact of employee AI usage on organizations. According to other scholars, although the AI usage in the workplace can simplify tasks, it may also lead to the loss of important personal resources, which could increase the sense of job alienation (Liu and Li, 2025). Moreover, AI technologies may lead to the disappearance of certain industries and jobs, triggering changes in the employment structure and increasing employment pressure (He, 2025). AI usage can exacerbate job alienation and expose its potential negative impacts, especially when the core task characteristics are highly substitutable (Liu and Li, 2025). These negative impacts undoubtedly pose challenges for both organizations and society. Therefore, in future research, this study will focus on the negative impacts of AI usage on employees, as well as the resulting organizational effects.

Second, many participants were aged 20 to 40 years. This age group is generally more receptive to accepting new technology and more likely to incorporate it into their work. However, older employees may have a lower level of technology acceptance. Therefore, future research should focus on organizations with a higher average employee age or a larger proportion of older employees to explore the acceptance and importance of new technologies among older employees and to analyze the actual impact on the organization based on the results.

Third, this study examined the role of AI usage in employee creativity within organizations in the context of Chinese SMEs. Considering the differences in geographic and cultural contexts, similar studies should be conducted to understand the situation in other countries. The impact of AI usage in different countries and cultural contexts has been explored through empirical analysis to determine whether the results are generalizable (Jin et al., 2023).

Fourth, this study identifies employee creativity as a key factor in the use of AI by employees. The importance of innovation has become more prominent (Chen K. J. et al., 2024). Therefore, future research should focus on the impact of employee AI usage on innovation behavior, innovation performance, and other related dimensions of innovation. It is important to expand the boundaries of research and fill the theoretical gaps in this area.

Fifth, although Amabile’s creativity scale has been widely recognized by researchers, considering the actual measurement context of employees in Chinese SMEs, this study selected a more suitable version of the scale. Future research may further explore the measurement equivalence of different versions of the creativity scale within the research model.

Sixth, the questionnaire employed in this study was not intentionally segmented, with all variables being self-reported by employees. This reliance on a single data source may lead to inflated correlations among variables, thereby increasing the potential for common method bias (CMB). To address this limitation, future research could refine the data collection process by, for instance, having employees assess organizational factors while organizational leaders evaluate employees’ attitudes, behaviors, and performance (Jin et al., 2023). Implementing such a multi-source data collection approach would likely enhance the validity and robustness of the study’s findings.

Finally, the cross-sectional design of this study may not fully clarify the different possible causal directions among the variables. Although our theoretical model assumes that AI usage influences employee creativity through work engagement, other plausible causal explanations may also exist. For example, employees with higher levels of creativity may be more engaged in their work and may also hold more open attitudes toward new technologies. Similarly, employees with higher technology acceptance may inherently possess stronger innovation tendencies, rather than these being merely a consequence of AI usage. Therefore, future research is encouraged to adopt longitudinal designs (e.g., cross-lagged panel analysis) or experimental methods to more rigorously examine the causal directions among the variables and to rule out the possibility of reverse causality. At a minimum, future studies should track changes in AI usage, work engagement, and employee creativity among the same group of employees at multiple time points to more deeply reveal the dynamic causal relationships among these variables.

## Data Availability

The datasets generated and/or analyzed during the current study are available from the corresponding author upon reasonable request. Requests to access the datasets should be directed to Xiu Jin, soohua1005, soohua1005@gachon.ac.kr.
